# Supplementary material for: Noninvasive electromyometrial imaging of human uterine maturation during term labor
Source: Nat Commun. 2023 Mar 14;14:1198. doi: 10.1038/s41467-023-36440-0 (PMC10015052; doi:10.1038/s41467-023-36440-0)
Supplement: Supplementary file 1 — Supplementary Information [file 41467_2023_36440_MOESM1_ESM.docx]

# Supplementary Information


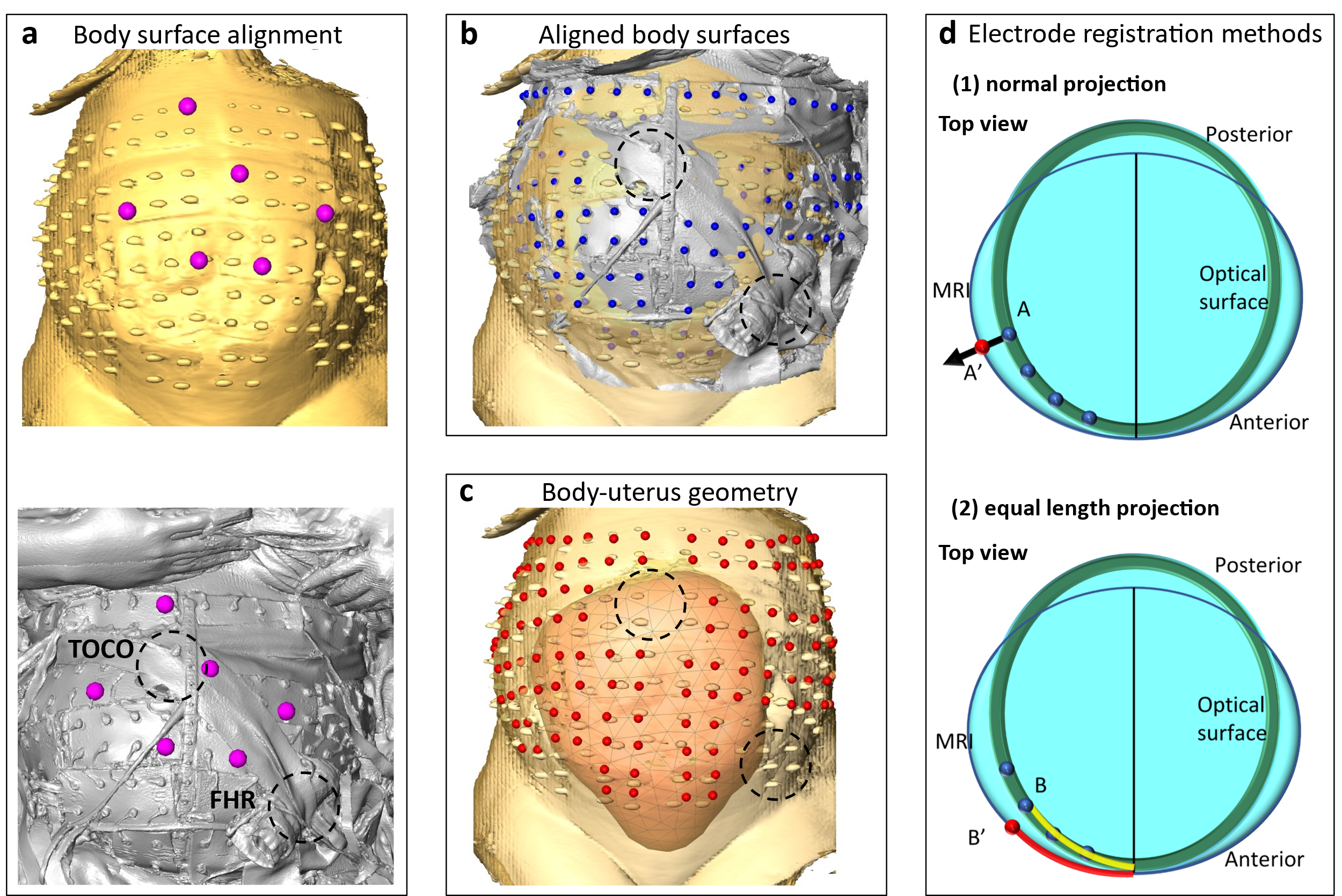


**Supplementary Fig.** **1 Schematic of geometry registration.** a, Body surface alignment. The top shows the body surface generated from the MR images (golden surface), with up to 192 markers. The bottom shows the body surface modeled from the optical 3D scanning (grey surface) wearing the electrode patches and clinical monitors (TOCO and FHR, labeled as black dashed circles). The alignment markers (purple dots) are defined on each surface for the calculation of the least-square rigid alignment relationship. b, Aligned body surfaces overlaying each other (yellow, MRI; grey, optical 3D scanning). The blue dots delineate electrode locations in optical 3D scanning. c, Body-uterus geometry, where the red dots represent electrode locations after registration. The pink triangulation mesh represents the uterine surface. d, The methods of electrode registration. The green circle represents the optical body surface, and the cyan oval represents the MRI body surface from the top view. The blue dots (A and B) represent the represent electrode locations on the optical body surface, and the red dots (A’ and B') represent the projected electrode locations on the MRI body surface. (1) In normal projection, the arrow represents the normal direction at A on the optical body surface. (2) In equal length projection, the yellow curve measures the surface distance from the vertical ruler to B on the optical body surface at the cross-section of the top view; the red curve measures that to B’ on the MRI body surface. The lengths of the two curves are kept equal. MRI, magnetic resonance imaging; 3D, three-dimensional; TOCO, tocodynamometer; FHR, fetal heart rate.


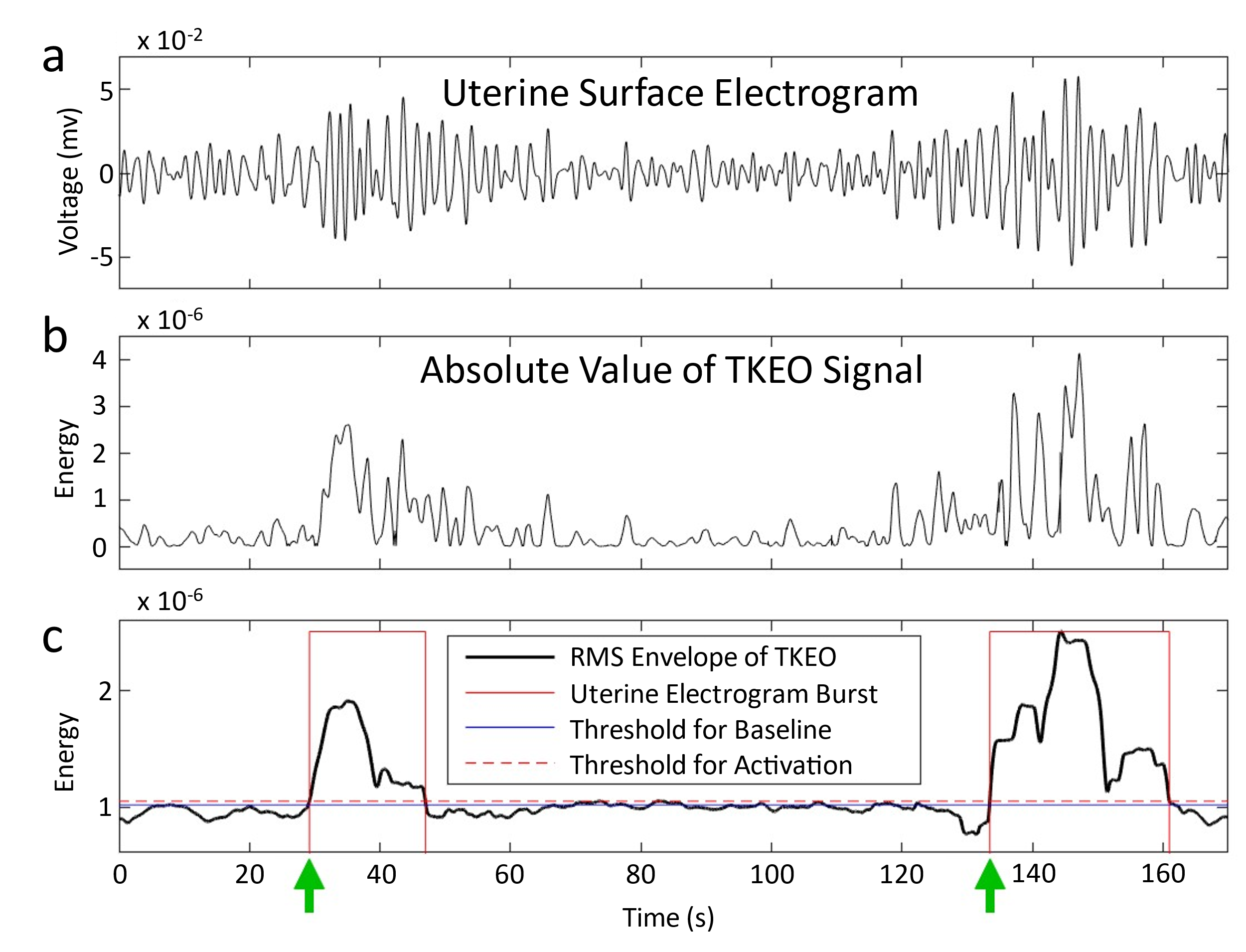


**Supplementary Fig. 2** Demonstration of uterine electrogram burst detection. a, A representative uterine electrogram. b, The rectified (absolute value) TKEO signal of the uterine electrogram. c, The detection of UEB. The black line denotes the RMS envelope of the rectified TKEO signal; the red line denotes the detected UEB; the blue line denotes the threshold of the baseline; the red dashed line denotes the threshold of the UEB; the green arrows denote the uterine electrical activation. TKEO, Teager-kaiser energy operator; RMS, root mean square; UEB, uterine electrogram burst.

The Matlab codes that generate the EMMI images. These codes are developed in Matlab 2019b. Please see the examples in **EMMI.m** and use the **Source Data file** for implementation. Email us with any questions, or comments: [zwen@wustl.edu](mailto:zwen@wustl.edu), [wangyong@wustl.edu](mailto:wangyong@wustl.edu)

# EMMI.m

%% EMMI - Source Codes

% To generate the figures with Source Data for

% Noninvasive Electromyometrial Imaging of Human Uterine Maturation During Term Labor

% Zichao Wen | zwen@wustl.edu | Jan. 04, 2023

SourceData_filename = 'SourceData.xlsx';

%% Fig. 2b - potential maps

% generate the potential map for a spefic time

% ======= manual input =======

mapTime = 29; % [s]

% ============================

bodyTime = readtable(SourceData_filename, ...

'Sheet', 'Fig2bcde_BodyPotentialMaps', ...

'Range','J2:AMT2',...

'ReadVariableNames', false);

bodyTime = table2array(bodyTime);

mapIdx = fix(interp1(bodyTime, find(bodyTime), mapTime));

% ------- load potential maps and geometry from Source Data files

% body surface

bodyPts = readtable(SourceData_filename, ...

'Sheet', 'Fig2bcde_BodyPotentialMaps', ...

'Range','B3:D162',...

'ReadVariableNames', false);

bodyTri = readtable(SourceData_filename, ...

'Sheet', 'Fig2bcde_BodyPotentialMaps', ...

'Range','E3:G318',...

'ReadVariableNames', false);

bodyMaps = readtable(SourceData_filename, ...

'Sheet', 'Fig2bcde_BodyPotentialMaps', ...

'Range','J3:AMT162',...

'ReadVariableNames', false);

% uterine surface

uterinePts = readtable(SourceData_filename, ...

'Sheet', 'Fig2bcde_UterusPotentialMaps', ...

'Range','B3:D322',...

'ReadVariableNames', false);

uterineTri = readtable(SourceData_filename, ...

'Sheet', 'Fig2bcde_UterusPotentialMaps', ...

'Range','E3:G638',...

'ReadVariableNames', false);

uterineTime = readtable(SourceData_filename, ...

'Sheet', 'Fig2bcde_UterusPotentialMaps', ...

'Range','J2:AMT2',...

'ReadVariableNames', false);

uterineMaps = readtable(SourceData_filename, ...

'Sheet', 'Fig2bcde_UterusPotentialMaps', ...

'Range','J3:AMT322',...

'ReadVariableNames', false);

% convert table to matrix

bodyMaps = table2array(bodyMaps);

bodyTri = table2array(bodyTri);

bodyPts = table2array(bodyPts);

uterineMaps = table2array(uterineMaps);

uterineTri = table2array(uterineTri);

uterinePts = table2array(uterinePts);

% plot body surface potential map

figure;

h_body = generate_potential_map(bodyMaps(:,mapIdx), bodyTri, bodyPts);

% plot body uterine potential map

figure;

h_uterus = generate_potential_map(uterineMaps(:,mapIdx), uterineTri, uterinePts);

%% Fig 3b - uterine eletrogram signals

% load uterine electrogram data from Source Data files

uterineEMGtable = readtable(SourceData_filename, ...

'Sheet', 'Fig3b_UterineEMG', ...

'PreserveVariableNames', true);

% plot

figure;

time_uterineEMG = table2array(uterineEMGtable(:,1));

for siteIdx = 1:5

% get EMG data

tmp_EMG = table2array(uterineEMGtable(:,siteIdx+1));

subplot(5,1,siteIdx);

plot(time_uterineEMG, tmp_EMG, 'color', 'k');

xlim([min(time_uterineEMG), max(time_uterineEMG)]);

ylim([-0.1, 0.1]);

xlabel('time (s)');

ylabel('voltage (mV)');

end

%% Fig. 3d - isochrone map and early activation map

% load uterine activation times from Source Data files

uterineActivation = readtable(SourceData_filename, ...

'Sheet', 'Fig3cde_IsochroneEarly', ...

'Range','I3:J322',...

'ReadVariableNames', false);

% uterine geometry

uterinePts = readtable(SourceData_filename, ...

'Sheet', 'Fig3cde_IsochroneEarly', ...

'Range','B3:D322',...

'ReadVariableNames', false);

uterineTri = readtable(SourceData_filename, ...

'Sheet', 'Fig3cde_IsochroneEarly', ...

'Range','E3:G638',...

'ReadVariableNames', false);

% convert table to matrix

uterineActivation = table2array(uterineActivation);

uterineTri = table2array(uterineTri);

uterinePts = table2array(uterinePts);

% plot isochrone map

activationTimes = uterineActivation(:,1);

h_iso = generate_isochrone_map(activationTimes, uterineTri, uterinePts);

% plot early activation map

earlyActivation = uterineActivation(:,2);

h_early = generate_early_activation_map(earlyActivation, uterineTri, uterinePts);

%% Fig. 3f - activation curve

% load uterine activation times from Source Data files

uterineActivation = readtable(SourceData_filename, ...

'Sheet', 'Fig3cde_IsochroneEarly', ...

'Range','I3:J322',...

'ReadVariableNames', false);

uterineActivation = table2array(uterineActivation);

% get activation curve

activationTimes = uterineActivation(:,1);

activationCurve = get_activation_curve(activationTimes);

% plot activation curve

figure;

hold on;

plot(activationCurve(:,1), activationCurve(:,2), 'Color', 'b', 'LineWidth', 2);

% the slope

t_min = min(activationCurve(:,1));

t_max = max(activationCurve(:,1));

r_min = min(activationCurve(:,2));

r_max = max(activationCurve(:,2));

plot([t_min, t_max], [r_min, r_max], '--', 'Color', 'k', 'LineWidth', 2)

xlim([0 40]);

ylim([0 50.5]);

xlabel('Time (s)');

ylabel('Activation Ratio (%)');

% ------ end of EMMI.m -------

# generate_early_activation_map.m

function h = generate_early_activation_map(earlyActivation, tri, pts)

%GENERATE_EARLY_ACTIVATION_MAP Summary of this function goes here

% get coordinates

x = pts(:,1);

y = pts(:,2);

z = pts(:,3);

earlyActivation(isnan(earlyActivation)) = -0.01;

% ------- figure setting -------

% plotting parameters

plotColorMap = jet;

plotColorMap(1,:) = [110 110 110]/255;

colorMapRange = [min(earlyActivation), max(earlyActivation)];

% figure configuration

h = figure('Name', 'isochrone map of activation',...

'units','inch',...

'outerposition',[5,5,7,2.5]);

tmpaxe = myaxes(1,4);

% plot isochrone map on the four views

for axe_id = 1:numel(tmpaxe)

axes(tmpaxe(axe_id));

% plot trisurf

trisurf(tri, x, y, z, earlyActivation,...

'FaceAlpha',1,...

'EdgeColor','none',...

'AmbientStrength', 0.4,...

'FaceColor','interp');

% change view

view([90*(axe_id-1), 0]);

axis equal vis3d off

% randering

colormap(plotColorMap);

caxis(gca, colorMapRange);

camlight;

lighting(tmpaxe(axe_id),'gouraud');

end

end

% ------ end of generate_early_activation_map.m -------

# generate_isochrone_map.m

function h = generate_isochrone_map(activationTimes, tri, pts)

%GENERATE_ISOCHRONE_MAP Summary of this function goes here

% get coordinates

x = pts(:,1);

y = pts(:,2);

z = pts(:,3);

% activation time - assign inactive sites with max+1

inactive_index = isnan(activationTimes);

if sum(inactive_index)>0

activationTimes(inactive_index) = max(activationTimes)+1;

end

% ------- figure setting -------

% plotting parameters

plotColorMap = flipud(jet);

plotColorMap(end,:) = [110 110 110]/255;

colorMapRange = [min(activationTimes), max(activationTimes)];

% figure configuration

h = figure('Name', 'isochrone map of activation',...

'units','inch',...

'outerposition',[5,5,7,2.5]);

tmpaxe = myaxes(1,4);

% plot isochrone map on the four views

for axe_id = 1:numel(tmpaxe)

axes(tmpaxe(axe_id));

% plot trisurf

trisurf(tri, x, y, z, activationTimes,...

'FaceAlpha',1,...

'EdgeColor','none',...

'AmbientStrength', 0.4,...

'FaceColor','interp');

% change view

view([90*(axe_id-1), 0]);

axis equal vis3d off

% randering

colormap(plotColorMap);

caxis(gca, colorMapRange);

camlight;

lighting(tmpaxe(axe_id),'gouraud');

end

end

% ------ end of generate_isochrone_map.m -------

# generate_potential_map.m

function h = generate_potential_map(potentialMap, tri, pts)

%GENERATE_POTENTIAL_MAP Summary of this function goes here

gIn.tri = tri;

gIn.pts = pts;

% ------- setting -------

maps = [0, 0, 1;

0, 1, 1;

152/255,251/255,152/255;

1, 1, 0;

1, 0, 0;

1, 0, 1;

1, 1, 1];

maprange = [-0.05, 0.05];

h = view3dmap(potentialMap, gIn);

view([0,0]);

colormap(maps);

camlight('left');

lighting('gouraud');

caxis(maprange);

end

% ------ end of generate_potential_map.m -------

# get_activation_curve.m

% Derive the activation curve for an isochrone map of uterine electrical activaiton

% "Noninvasive Electromyometrial Imaging of Human Uterine Maturation During Term Labor"

% Zichao Wen | zwen@wustl.edu | Dec. 4, 2022

% input: activationTimes [double] - activation times from spreadsheet (s)

% output: activationCurve [double] - the derived activation curve

% 1st column, timestamps when the activation ratio change (s)

% 2nd column, corresponding activation ratios (%)

function activationCurve = get_activation_curve(activationTimes)

% count the number of uterine sites

N = numel(activationTimes);

% exclude inactive (NaN) sites

actT_valid = activationTimes(~isnan(activationTimes));

% unique ativation times as timestampes when the activation ratio change (s)

activationCurve_time = unique(actT_valid);

% derive the count for eqch unique activation time using histogram bin counts

actT_edges = [activationCurve_time-1e-5; activationCurve_time(end)+1e-5];

actT_counts = histcounts(actT_valid, actT_edges);

% calculate the activation ratios (%)

activationCurve_ratio = cumsum(actT_counts(:))/N*100;

% output

activationCurve_time = [0; activationCurve_time(:)];

activationCurve_ratio = [0; activationCurve_ratio(:)];

activationCurve = [activationCurve_time, activationCurve_ratio];

end

% ------ end of get_activation_curve.m -------

# myaxes.m

function hAx = myaxes(varargin)

% arrayH = MYAXES(numRow, numCol)

% Create axes array

% Output a colomn vector of handles indexing the axeses in row-first order

% If numCol > 0, create (numRow * numCol) axeses in [num_row, num_colown] shape

% If numRow <= 0, axeses (numRow) axeses most squared shape

%

% arrayH = MYAXES(..., blankRow)

% arrayH = MYAXES(..., blankRow, blankCol)

% blankRow = [blankBottom, blankAbove, offsetBottom]

% blankCol = [blankLeft, blankRight, offsetLeft]

% If numel(blankRow) < 3, then offsetBottom = 0

% If numel(blankRow) == 1, then blankRow = blankBottom = blankAbove

% Same for blankCol

%

% arrayH = MYAXES(..., propName, propValue)

% see mysetarray

%

% arrayH = MYAXES(hFig, ...)

% if hFig is not specified, use current figure

if (isscalar(varargin{1}) && isa(varargin{1}, 'matlab.ui.Figure'))

hFig = varargin{1};

if (~isvalid(hFig))

throw(MException('myError:myError', 'Trying to use a cleared figure'));

end

varargin = varargin(2:end);

else

hFig = gcf;

end

numRow = varargin{1};

numCol = varargin{2};

varargin = varargin(3:end);

if (numCol <= 0)

numAll = numRow;

numCol = ceil(sqrt(numRow));

% numCol = floor(sqrt(numRow));

numRow = ceil(numRow / numCol);

else

numAll = numRow * numCol;

end

hAx = repmat(gca(hFig), 1, numAll);

delete(findall(hFig, 'type', 'axes'));

% %%%%%%%%%%%%%%%%%%%%%%%%%%%%%%%%%%%%%%%%%%%%%%%%%%%%%%%%%%%%%%%%%%%%%

% calculate axes size

% %%%%%%%%%%%%%%%%%%%%%%%%%%%%%%%%%%%%%%%%%%%%%%%%%%%%%%%%%%%%%%%%%%%%%

R_left = 0.05/numRow; R_right = 0.05/numRow; R_base = 0.01;

C_left = 0.05/numCol; C_right = 0.05/numCol; C_base = 0.01;

if (~isempty(varargin) && isnumeric(varargin{1}))

R_left = varargin{1}(1);

if (numel(varargin{1}) <= 1)

R_right = R_left;

else

R_right = varargin{1}(2);

end

if (numel(varargin{1}) > 2)

R_base = varargin{1}(3);

end

varargin = varargin(2:end);

end

if (~isempty(varargin) && isnumeric(varargin{1}))

C_left = varargin{1}(1);

if (numel(varargin{1}) <= 1)

C_right = C_left;

else

C_right = varargin{1}(2);

end

if (numel(varargin{1}) > 2)

C_base = varargin{1}(3);

end

varargin = varargin(2:end);

end

widthX = (1-R_base)/numRow - R_left - R_right;

widthY = (1-C_base)/numCol - C_left - C_right;

% %%%%%%%%%%%%%%%%%%%%%%%%%%%%%%%%%%%%%%%%%%%%%%%%%%%%%%%%%%%%%%%%%%%%%

% create axes

% %%%%%%%%%%%%%%%%%%%%%%%%%%%%%%%%%%%%%%%%%%%%%%%%%%%%%%%%%%%%%%%%%%%%%

for indA = 1:numAll

[indY, indX] = ind2sub([numCol, numRow], indA);

posX = R_base + R_left + (1 - R_base) * (numRow-indX)/numRow;

posY = C_base + C_left + (1 - C_base) *(indY-1)/numCol;

hAx(indA) = axes('position', [posY, posX, widthY, widthX]);

end

% %%%%%%%%%%%%%%%%%%%%%%%%%%%%%%%%%%%%%%%%%%%%%%%%%%%%%%%%%%%%%%%%%%%%%

% set properties

% %%%%%%%%%%%%%%%%%%%%%%%%%%%%%%%%%%%%%%%%%%%%%%%%%%%%%%%%%%%%%%%%%%%%%

mysetarray(hAx, varargin{:});

end

% ------ end of myaxes.m -------

# mysetarray.m

% MYSETARRAY(arrayH, propName, propValue) Set properties for object array

%

% If propValue is a cell array, it should be the same length as arrayH and contains

% values for each element of arrayH.

%

% If propName starts with "func", then the following is the name of the function with

% two inputs and <value> is the second input

% mysetarray(ax, 'func_hold', 'on') - hold(ax, 'on')

function mysetarray(hdl, varargin)

numH = numel(hdl);

for k = 1:2:length(varargin)

propName = varargin{k};

propVal = varargin{k+1};

if (~iscell(propVal)) % non-cell : the sampe propVal for all

propVal = repmat({propVal}, 1, numH);

elseif (numel(propVal) == 1) % cell, only 1 element : the sampe propVal for all

propVal = repmat(propVal, 1, numH);

elseif (numel(propVal) ~= numH)

throw(MException('myError:myError', sprintf('Wrong axes property: %s', propName)));

end

for indH = 1:numH

if (~isvalid(hdl(indH)))

continue;

end

if (strncmpi(propName, 'func_', 5))

if (isempty(propVal{indH}))

feval(lower(propName(6:end)), hdl(indH));

elseif (iscell(propVal{indH}))

feval(lower(propName(6:end)), hdl(indH), propVal{indH}{:});

else

feval(lower(propName(6:end)), hdl(indH), propVal{indH});

end

else

set(hdl(indH), propName, propVal{indH});

end

end

end

end

% ------ end of mysetarray.m -------

# view3dmap.m

function h = view3dmap(distribution, geometry, varargin)

% vis3dmap(distribution, geometry)

% INPUT: - distribution: numPts * 1;

% - geometry: struct('tri','pts')

% Full syntax: [H] = view3dmap(distribution, geometry, <opt>)

% OPTIONAL INPUT;

% - 'faceA', <float> - option of transparency of face

% - Default: 1; (0,1)

% - 'ax', <axes> - option of axes

% - Default: current axes

% - 'view', <obj> - option of view

% - Default: [0,0]

% Hui Wang

% wang.hui@wustl.edu

%% Input parsing

p = inputParser;

addRequired(p,'distribution');

addRequired(p,'geometry');

addOptional(p,'faceA',1);

addOptional(p,'ax',gca);

addOptional(p,'view',[0,0]);

parse(p,distribution,geometry,varargin{:})

%%

faceA = p.Results.faceA;

axes(p.Results.ax);

h = trisurf(geometry.tri,...

geometry.pts(:,1),geometry.pts(:,2),geometry.pts(:,3),...

distribution.*ones(size(geometry.pts,1),1),...

'FaceAlpha',faceA,'EdgeColor','none',...

'EdgeAlpha',0.5,'AmbientStrength',0.4,'FaceColor','interp');

% set(h, 'parent',p.Results.ax);

colormap(p.Results.ax, jet);

% colorbar

axis(p.Results.ax, 'equal', 'off', 'vis3d');

view(p.Results.ax, p.Results.view);

% camlight; lighting(gca,'gouraud');

End

% ------ end of view3dmap.m -------
