# Supplementary material for: Noninvasive electromyometrial imaging of human uterine maturation during term labor
Source: Nat Commun. 2023 Mar 14;14:1198. doi: 10.1038/s41467-023-36440-0 (PMC10015052; doi:10.1038/s41467-023-36440-0)
Supplement: Supplementary file 2 — Reporting Summary [file 41467_2023_36440_MOESM2_ESM.pdf]

nature portfolio

Corresponding author(s): Yong Wang
Last updated by author(s): Dec 12, 2022

Reporting Summary

Nature Portfolio wishes to improve the reproducibility of the work that we publish. This form provides structure for consistency and transparency in reporting. For further information on Nature Portfolio policies, see our Editorial Policies and the Editorial Policy Checklist.

Statistics

For all statistical analyses, confirm that the following items are present in the figure legend, table legend, main text, or Methods section.

n/a	Confirmed	
☒	☐	The exact sample size (*n*) for each experimental group/condition, given as a discrete number and unit of measurement
☒	☐	A statement on whether measurements were taken from distinct samples or whether the same sample was measured repeatedly
☒	☐	The statistical test(s) used AND whether they are one- or two-sided<br>*Only common tests should be described solely by name; describe more complex techniques in the Methods section.*
☒	☐	A description of all covariates tested
☒	☐	A description of any assumptions or corrections, such as tests of normality and adjustment for multiple comparisons
☐	☒	A full description of the statistical parameters including central tendency (e.g. means) or other basic estimates (e.g. regression coefficient) AND variation (e.g. standard deviation) or associated estimates of uncertainty (e.g. confidence intervals)
☒	☐	For null hypothesis testing, the test statistic (e.g. *F*, *t*, *r*) with confidence intervals, effect sizes, degrees of freedom and *P* value noted<br>*Give P values as exact values whenever suitable.*
☒	☐	For Bayesian analysis, information on the choice of priors and Markov chain Monte Carlo settings
☒	☐	For hierarchical and complex designs, identification of the appropriate level for tests and full reporting of outcomes
☒	☐	Estimates of effect sizes (e.g. Cohen's *d*, Pearson's *r*), indicating how they were calculated

Our web collection on statistics for biologists contains articles on many of the points above.

Software and code

Policy information about availability of computer code

Data collection	Artec Studio 12 is used for optical 3D scanning. ActiView 8.09 is used for bioelectrical recording.
Data analysis	Artec Studio 12 is used for building surface model from optical 3D scanning. Thermo Scientific Amira Software 6.4 is used for MRI segmentation, geometry registration and landmark placement. Matlab 2019b is used for data processing.

For manuscripts utilizing custom algorithms or software that are central to the research but not yet described in published literature, software must be made available to editors and reviewers. We strongly encourage code deposition in a community repository (e.g. GitHub). See the Nature Portfolio guidelines for submitting code & software for further information.

Data

Policy information about availability of data

All manuscripts must include a data availability statement. This statement should provide the following information, where applicable:

- Accession codes, unique identifiers, or web links for publicly available datasets
- A description of any restrictions on data availability
- For clinical datasets or third party data, please ensure that the statement adheres to our policy

We have declared that the data supporting the findings of this study are available within the paper. The geometry, electromyography, uterine electrical activation isochrone map, and early activation map data generated in this study are provided in the Source Data file.

Human research participants

Policy information about studies involving human research participants and Sex and Gender in Research.

Reporting on sex and gender	This study applies to only females.
Population characteristics	10 term subjects are presented, among which 5 are nulliparous and 5 multiparous. The ages of the patients range from 18 to 35 years old. The cervical dilation of the patients during the EMMI mapping was in the range of 3.5 to 9.5 cm.
Recruitment	Nulliparous and multiparous patients were recruited when they were in person after a clinic appointment without bias. The study was explained, and those who are willing to participate underwent informed consent.
Ethics oversight	This study was approved by the Washington University Institutional Review Board (No. 201612140).

Note that full information on the approval of the study protocol must also be provided in the manuscript.

Field-specific reporting

Please select the one below that is the best fit for your research. If you are not sure, read the appropriate sections before making your selection.

☒ Life sciences ☐ Behavioural & social sciences ☐ Ecological, evolutionary & environmental sciences

For a reference copy of the document with all sections, see nature.com/documents/nr-reporting-summary-flat.pdf

Life sciences study design

All studies must disclose on these points even when the disclosure is negative.

Sample size	5 nulliparous and 5 multiparous women underwent multichannel body surface electrical recording up to 1 hour total. This pilot study focuses on the development and initial application of the human EMMI system. And we imaged 10 subjects to demonstrate the clinical feasibility and initial findings. No statistical analysis is involved in this study.
Data exclusions	Poor contact signals and motion artifacts were excluded from the electrical data.
Replication	The study protocol is replicable. But the same data may not be acquired because the events of labor and uterine contractions of specific patients are not replicable.
Randomization	Not relevant to this study. This pilot study focuses on the development and initial application of the human EMMI system. No statistical analysis is involved in this study.
Blinding	Not relevant to this study. This pilot study focuses on the development and initial application of the human EMMI system. No statistical analysis is involved in this study.

Reporting for specific materials, systems and methods

We require information from authors about some types of materials, experimental systems and methods used in many studies. Here, indicate whether each material, system or method listed is relevant to your study. If you are not sure if a list item applies to your research, read the appropriate section before selecting a response.

Materials & experimental systems

n/a	Involved in the study	
☒	☐	Antibodies
☒	☐	Eukaryotic cell lines
☒	☐	Palaeontology and archaeology
☒	☐	Animals and other organisms
☒	☐	Clinical data
☒	☐	Dual use research of concern

Methods

n/a	Involved in the study	
☒	☐	ChIP-seq
☒	☐	Flow cytometry
☒	☐	MRI-based neuroimaging
